# Do pseudoxanthoma elasticum patients have higher prevalence of kidney stones on computed tomography compared to hospital controls?

**DOI:** 10.1007/s10157-023-02405-2

**Published:** 2023-10-14

**Authors:** Iris M. Harmsen, Madeleine Kok, Jonas W. Bartstra, Pim A. de Jong, Wilko Spiering, Wouter Foppen

**Affiliations:** 1 Department of Vascular Medicine, University Medical Center Utrecht, Utrecht University, P.O. Box 85500, 3508 GA Utrecht, The Netherlands; 2 Department of Radiology, University Medical Center Utrecht, Utrecht University, Utrecht, the Netherlands

**Keywords:** Pseudoxanthoma elasticum, PXE, ABCC6, Urolithiasis, Kidney stones, Computed tomography, CT

## Abstract

**Background:**

Pseudoxanthoma elasticum (PXE) is an autosomal recessive disease characterized by diminished inorganic plasma pyrophosphate (PPi), a strong calcification inhibitor. In addition to more typical calcification of skin, retina and arterial wall a diminished plasma PPi could lead to other ectopic calcification, such as formation of kidney stones.

**Objective:**

To compare the prevalence of kidney stones between PXE patients and hospital controls on computed tomography (CT).

**Method:**

Low-dose CT images of PXE patients and controls were assessed by one radiologist, who was blinded for the diagnosis PXE. The number of kidney stones, and the size of the largest stone was recorded. Odds ratios (ORs) for having kidney stone were calculated using multivariable adjusted logistic regression.

**Results:**

Our study comprised 273 PXE patients and 125 controls. The mean age of PXE patients was 51.5 ± 15.9 years compared to 54.9 ± 14.2 in the control group (*p* = 0.04) and PXE patients more often were women (63 vs. 50%, *p* = 0.013). The prevalence of kidney stones on CT was similar: 6.9% in PXE patients, compared to 5.6% in controls (*p* = 0.6). In the multivariate analysis adjusting for age and sex, there was no significantly higher odds for PXE patients on having stones, compared to controls: OR 1.48 (95% CI 0.62–3.96).

**Conclusion:**

There is no significant difference in the prevalence of incidental kidney stones on CT in PXE patients versus controls.

## Introduction

Pseudoxanthoma elasticum (PXE) is an autosomal recessive disease [1] caused by pathogenic mutations in the *ABCC6* gene. According to the currently accepted hypothesis regarding the pathophysiology, PXE is a metabolic disease where a relative lack of inorganic plasma pyrophosphate (PPi) is central [2, 3]. PPi is a strong calcification inhibitor [4, 5], and diminished circulating PPi levels explains the three main PXE symptoms: calcification in the elastic fibers in the skin, calcification in Bruch’s membrane of the retina and calcification of the medial layer of the arterial wall [1, 6].

It is possible that a systemic diminished plasma PPi in PXE would increase the risk of ectopic calcification in other organs. In fact, other diseases that are characterized by low plasma PPi, such as general arterial calcification of infancy (GACI) and arterial calcification due to CD73 deficiency (ACDC) also exhibit different symptoms [7]. One of the most common and well-known ectopic calcifications are kidney stones. Low levels of urinary PPi have been found to accelerate urinary crystallization in laboratory settings [8, 9]. Therefore, kidney stones have increasingly been suggested as a manifestation of PXE. Earlier studies have found heterogeneous prevalence’s ranging from 1.7 to 40% [10–13]. However, these findings might be biased due to lack of an adequate control group, and the use of a questionnaire, and need to be substantiated.

A more unbiased way to identify if kidney stones are a hallmark of PXE, is by assessing CT scans that have been performed in the regular PXE-workup. Incidental kidney stones are found in around 8% of abdominal CT scans in unselected populations [14]. We aimed to objectively measure the prevalence of kidney stones on CT in PXE patients and representative hospital controls. We hypothesize that if PXE patients have significantly higher risk of kidney stone, we will see a higher prevalence of incident kidney stones on CT compared with controls.

## Methods

### Study population

Patients were recruited from the Dutch Expertise Center for Pseudoxanthoma elasticum (DECP) in the University Medical Center Utrecht. All PXE patients in the study have signed their written informed consent to collect and use data. For the current study, data from patients were used with a confirmed diagnosis according the Plomp criteria [6], and if a CT scan was available from regular work-up to assess vascular calcification. Controls were consecutive subjects that received a fluorodeoxyglucose (FDG) positron emission tomography (PET) CT in our hospital between June 2011 July 2019, patients with suspicion of vasculitis or endocarditis were excluded, the main indication was melanoma staging. Because of the retrospective nature of this study, and the absence of any research interventions in the control population, the medical ethics committee Utrecht, waived the need for informed consent for the use of this imaging (protocol number 19/470).

### Imaging protocol

All subjects underwent low-dose (< 3 mSv for a 70 kg adult), full-body, non-contrast, CT scans with slice thickness of 1–2 mm (reconstructed at 5 mm × 4 mm increments) on either Siemens Biograph 40 (Erlangen, Germany) or Philips Brilliance (Best, The Netherlands). CT scans were made at variable kVp and mAs dependent on the subjects body weight.

### Measurements

The thin-slice images were viewed by a trained radiologist (WF), who measured the number of stones per kidney, the size of the largest stone (in mm) and the HU value of the largest kidney stone, blinded for patient status (PXE or control patient). From all subjects age and sex were available.

### Statistical analysis

Continuous data are presented with the mean and standard deviation for Gaussian distributed values, and with the median and interquartile range for non-Gaussian distributed values. The differences in cases and controls regarding age and sex were tested with, respectively, *T*-test and Chi-square test. Logistic regression models were used to control for potential differences in sex and age between cases and controls. A sensitivity analysis was performed with inverse probability weighting using propensity scores. We calculated propensity scores for having PXE based on age and sex, using logistic regression. Statistical analysis was performed with R-software version 4.0.3 (R foundation for Statistical Computing, Vienna, Austria).

## Results

We studied 269 PXE patients and 125 controls. Baseline characteristics are reported in Table 1. The mean ± SD age of PXE patients was 51.5 ± 14.2 years compared to 54.9 ± 15.9 years in the control group (*p* = 0.04). PXE patients more often were women (63 vs 50%, *p* = 0.01).

**Table 1:** Baseline characteristics of PXE patients and controls

	Controls	PXE	*p*
	*n* = 125	*n* = 269	
Age (years), mean ± SD	54.86 ± 15.9	51.48 ± 14.2	0.04
Females, *n* (%)	62 (50)	171 (63)	0.01
Any stone, *n* persons (%)	7 (6)	19 (7)	0.74
Number of stones, *n* (%)			
1	5 (4.0)	12 (4.5)	
2	1 (0.8)	1 (0.4)	
3	0 (0.0)	3 (1.1)	
4	0 (0.0)	1 (0.4)	
5	1 (0.8)	1 (0.4)	
11	0 (0.0)	1 (0.4)	
Kidney stone size (mm), median [IQR]	5.0 [3.5, 5.5]	5.0 [3.0, 6.5]	0.91
HU kidney stone, mean [IQR]	1024.00 [559.50, 1068.00]	1214.50 [980.00, 1660.00]	0.06

*HU* houndsfield units

CT scans were all of sufficient quality and there were no missing values in the amount and size of kidney stones. There were three PXE patients where the HU value was not possible to measure because the kidney stones were too small. These subjects were excluded from the mean HU value.

There were 19 PXE patients that had at least one stone (7% (95% CI 4–10%)), versus 7 patients in the control group (6% (95% CI 2–10%), *p* = 0.7). The distribution of number of kidney stones is displayed in Table 1. The median [IQR] size of the kidney stones was 5.0 mm [3–6.5] for cases as well as controls (5.0 mm [3.5–5.5], *p* = 0.91). The median [IQR] HU value of the largest kidney stone was 1024 [560–1068] in controls and 1215 [980–1660] in PXE patients (*p* = 0.06).

A logistic regression was performed with the diagnosis PXE as a determinant of kidney stones. In the univariate model, the odds ratio for having a kidney stone on CT for PXE patients versus controls was 1.28 (95% CI 0.55–3.36). The age and sex adjusted odds ratio was 1.48 (95% CI 0.62–3.96) (Table 2).

**Table 2:** Multivariate logistic regression analysis of presence of any stone on CT-imaging

	*N*	Bèta	St. error	*p*	Odds ratio (95% CI interval)
Diagnosis					
Controls	125	Reference			
PXE patients	267	0.39	0.47	0.40	1.48 (0.62–3.96)
Age					
One year increase		0.02	0.02	0.07	1.03 (1.00–1.06)
Sex					
Men	161	Reference			
Women	233	−0.18	0.41	0.66	−1.20 (−0.37 to −1.91)

The balance after inverse probability weighting was excellent: standardized mean differences (SMD) between PXE and controls were 0.008 and 0.005, respectively, for age and sex. The odds ratio from the logistic regression using inverse probability weighting was 1.38 (95% CI 0.77–2.48).

## Discussion

This is the first study to objectively assess the prevalence of kidney stones in PXE-patients on CT scan with a representative control group. There was no statistically significant difference in the prevalence of kidney stones on CT between PXE patients and controls.

Strengths of our study include the availability of CT scans for a large population of PXE patients and the unbiased manner of assessing incidental kidney stones: with the use of CT scans in both cases and controls, viewed by a radiologist blinded for the diagnosis PXE. Another strength is that we were able to correct at least for imbalances in age and sex between cases and controls.

A limitation of this study is that because of the retrospective nature of this study, and the anonymous data handling in the control group, we were not able to collect more information from controls because of privacy reasons. We were, therefore, unable to collect data on symptoms of kidney stone disease at the time of the CT scan. Another limitation is that, even though we have a large group of PXE patients available, we have limited controls study. Together with the low prevalence of kidney stones, this leads to diminished power. However, the only previously reported patient-control comparison showed a very large difference in prevalence of kidney stones between PXE-patients and controls (25 vs 9%). Our sample size would have been large enough to detect a significant difference of this magnitude. PXE-patients were slightly younger and more often female, both increasing age and male sex are a risk factor for kidney stone development [15]. Because of this, we have calculated the age and sex adjusted odds ratio for PXE on the presence of a kidney stone on CT: 1.48 (95% CI 0.62–3.96), pointing out that even after adjusting for age and sex there is still no statistical significant difference in the prevalence of kidney stones on CT images between cases and controls. The sensitivity analysis with probability weighting did not alter this conclusion 1.38 (95% CI 0.77–2.48).

Previous literature has reported on the prevalence of kidney stones in PXE patients, but the findings are heterogeneous [10–13]. Two of these studies have specifically tried to assess the relationship between PXE and kidney stones, with the use of a questionnaire [11, 16]. Ralph et al. reported a kidney stone prevalence of 25% in PXE populations, which they compared to the general population estimate of 9.1% [11]. Letavernier et al. [13], found that 39.8% of the 113 respondents had a kidney stone in their medical history. Even though they have not included a control group in this study, the prevalence is much higher than the general European and French population: 5–10% [17].

Contrary to these results stand the studies by Legrand et al. [12] and Boraldi et al. [10], who report a prevalence of kidney stones in PXE patients of, respectively, 12.8 and 1.7%, more in the range of the expected prevalence in the general European population. The studies were both cross-sectional assessments of the phenotypical features in the PXE populations, both authors do not state specifically how the prevalence of kidney stone was assessed.

Our results show that there is no significant difference between cases and controls in the prevalence of incidental kidney stones using visibility of stones on CT images as objective measure. The prevalence that was found in both the PXE and control group is also comparable with that of the prevalence of incidental kidney stones in the general population [18]. So, even though life-time prevalence of kidney stones and the prevalence of incidental kidney stones are not suitable for a direct comparison, our study is most in line with Legrand et al. [12] and Boraldi et al. [10].

The question remains why our conclusion does not match the conclusions of Ralph et al. [11] and Letavernier et al. [13]. The answer is likely found in the different methods of assessment: both these studies have used questionnaires to assess the prevalence of kidney stones. Questionnaires are known to be prone to bias[19]. There are several ways how this could lead to bias in these studies: first, PXE patients who have had a kidney stone, are more likely to answer such a questionnaire, and therefore researchers could have found a higher prevalence. Another point is that the questionnaire that both studies used, did not specifically ask for any proof that a (presumable) renal colic, was in fact caused by a kidney stone. Thirdly, they did not specify if the kidney stone was symptomatic, or if it could also have been an incidental finding on radiology. Since PXE patients are under the care of different medical specialists, and imaging is sometimes routinely executed, the chance of finding an incidental kidney stone that maybe would not have led to a renal colic is much higher than in control groups.

Especially the prevalence of kidney stones in the study of Letavernier et al. was very high. Possibly they have unintentionally identified a very specific subgroup of PXE patients that also has a higher risk of kidney stones, in addition to PXE, due to a common genetic, dietary or geographic predisposition.

It is important to identify if PXE patients exhibit more manifestations of their disease than what we have discovered up until now. First of all because we want to give the PXE patient the most accurate prognosis of what the disease PXE entails, secondly, the type and location of the symptom in question might be crucial in the unraveling of the pathophysiology of PXE. PXE patients exhibit lower PPi levels compared to controls, and these are believed, but not yet proven, to be related to the severity of symptoms [5, 20, 21]. Moreover, it is not yet clear how the *ABCC6* mutations work in lowering the PPi levels. The current hypothesis is that the ABCC6 transporter is involved in the transportation of ATP from the hepatocyte to the plasma [5], where ATP is then a substrate for ENPP1, the enzyme that converts it to PPi and AMP [4, 22]. The reason this is emphasized here, is that other (genetic) disease also can exhibit low PPi, through other mechanisms (ACDC [23], ANKH inorganic pyrophosphate transport regulator (ANKH) related disease [24, 25], GACI [26, 27]), and there is overlap in some symptoms, but other symptoms are unique. This gives us more insight in the regulation and metabolism of PPi and the effect on different tissues throughout the body. Our results seem to suggest that PXE is not inherently related to the formation of kidney stones, suggesting that PPi may be regulated by other enzymes in and around the kidney [28, 29], and that calcification does not solely rely on the presence of functional ABCC6 transporters. Moreover, there are many calcifying inhibitory regulations in the kidney, of which PPi is just one [15].

## Conclusion

In conclusion, we found no evidence for differences in the prevalence of kidney stones on CT images in PXE patients versus controls, when corrected for age and sex. This study contradicts the conclusions of previous studies that found a higher prevalence of kidney stones disease in PXE patients through questionnaires.

## Data Availability

The datasets used and/or analysed during the current study are available from the corresponding author on reasonable request.
